# Supplementary material for: Insight into the Molecular Signature of Skeletal Muscle Characterizing Lifelong Football Players
Source: Int J Environ Res Public Health. 2022 Nov 28;19(23):15835. doi: 10.3390/ijerph192315835 (PMC9740844; doi:10.3390/ijerph192315835)
Supplement: Supplementary file 1 [file ijerph-19-15835-s001.zip › Figure S1.pdf]

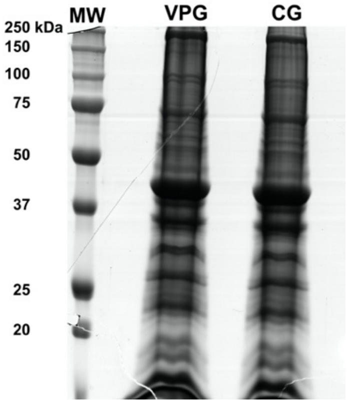


**Figure S1.** SDS-PAGE analysis of proteins in skeletal muscle from veteran football players (VPG) versus untrained subjects (CG). MW: molecular weight protein standard.
