# Supplementary material for: Insight into the Molecular Signature of Skeletal Muscle Characterizing Lifelong Football Players
Source: Int J Environ Res Public Health. 2022 Nov 28;19(23):15835. doi: 10.3390/ijerph192315835 (PMC9740844; doi:10.3390/ijerph192315835)
Supplement: Supplementary file 1 [file ijerph-19-15835-s001.zip › Table S1.pdf]

Table S1. Metabolite abbreviations and extended names

C0, free carnitine
C2, Acetylcarnitine
C3 Propionylcarnitine
C3, Propionylcarnitine
C4, Butyrylcarnitine
C5, Isovalerylcarnitine
C6, Hexanoylcarnitine
C8, Octanoylcarnitine/Caprylylcarnitine
C10, Decanoylcarnitine/Caprylcarnitine
C12, Dodecanoylcarnitine/Laurylcarnitine
C14, Tetradecanoylcarnitine/ Myristylcarnitine
C16, Hexadecanoylcarnitine/Palmitoylcarnitine
C18, Octadecanoylcarnitine/Stearylcarnitine
C5:1, Tiglylcarnitine/3-Methyl-crotonylcarnitine
C6:1, Hexenoylcarnitine
C8:1, Octenoylcarnitine
C10:1, Decenoylcarnitine
C10:2, Decadienoylcarnitine
C12:1, Dodecenoylcarnitine
C14:1; Tetradecenoylcarnitine
C14:2, Tetradecadienoylcarnitine
C16:1; Hexadecenoylcarnitine
C18:1; Octadecenoylcarnitine
C18:2; Octadecadienoylcarnitine
C4OH, 3-Hydroxybutyrylcarnitine
C5OH, 3-Hydroxyisovalerylcarnitine/3-hydroxy-2 methylbutyrylcarnitine
C6OH, 3-Hydroxyhexanoylcarnitine
C12OH, 3-Hydroxydodecanoylcarnitine
C14OH, 3-Hydroxytetradecanoylcarnitine
C16OH, 3-Hydroxyhexadecanoylcarnitine
C16:1OH, 3-Hydroxyhexadecenoylcarnitine
C18:1OH, 3-Hydroxyoctadecenoylcarnitine
C3DC, Malonylcarnitine
C4DC, Methylmalonilcarnitine
C5DC, Glutarylcarnitine
C6DC, Methylglutarylcarnitine
C8DC, Octanedioylcarnitine
C10DC, Decanedioylcarnitine
Ala, Alanine
Val, Valine
Xle, Leucin or Isoleucine
Met, Methionine
Phe, Phenylalanine
Tyr, Tyrosine

Asp, Aspartic Acid
Glu, Glutamic Acid
Gly, Glycine
Orn, Ornithine
Cit, Citrulline
Arg, Arginine
