# Supplementary material for: Insight into the Molecular Signature of Skeletal Muscle Characterizing Lifelong Football Players
Source: Int J Environ Res Public Health. 2022 Nov 28;19(23):15835. doi: 10.3390/ijerph192315835 (PMC9740844; doi:10.3390/ijerph192315835)
Supplement: Supplementary file 1 [file ijerph-19-15835-s001.zip › Table S2.pdf]

Table S2. Amino acid concentrations (microM) in muscle sample

	Ala	Val	Xle	Met	Phe	Tyr	Asp	Glu	Gly	Orn	Cit	Arg
**CG_1**	394.784	77.815	94.564	20.915	22.602	23.182	155.462	3187.066	615.114	42.237	35.753	101.08
**CG_2**	340.077	77.276	101.373	18.6	23.468	24.513	159.31	3329.234	620.992	42.088	35.277	105.381
**CG_3**	420.516	75.26	104.473	20.457	24.326	26.629	139.611	3212.395	617.464	42.773	35.961	104.984
**CG_4**	820.262	90.58	166.104	24.111	33.794	43.674	215.797	3166.61	869.132	95.277	67.432	183.704
**CG_5**	748.305	90.986	172.899	23.971	32.681	38.462	193.162	2910.959	861.941	90.922	69.67	187.384
**CG_6**	779.923	87.189	174.156	23.584	33.825	43.558	206.276	2711.098	861.665	93.271	67.824	185.209
**CG_7**	574.979	86.273	133.257	17.29	31.12	32.63	127.353	2094.326	559.988	70.196	39.901	116.583
**CG_8**	549.56	92.814	145.687	19.394	30.209	35.336	127.413	1974.643	560.031	69.501	40.055	115.881
**CG_9**	559.815	82.408	133.393	19.357	31.683	34.655	131.415	1868.448	555.33	68.107	40.046	115.323
**VPG_1**	621.636	80.132	129.015	19.47	31.665	35.135	75.869	2697.902	725.046	105.18	42.263	190.705
**VPG_2**	642.09	85.301	141.875	22.468	33.105	34.822	76.829	2772.18	716.182	108.12	41.951	192.694
**VPG_3**	635.031	83.407	133.85	21.723	33.438	36.802	76.196	2748.37	718.658	108.63	42.544	187.239
**VPG_4**	817.248	124.757	185.818	35.513	36.848	40.883	197.335	2657.836	938.453	100.91	22.544	163.089
**VPG_5**	753.827	124.595	194.63	33.525	38.173	37.472	195.66	2477.66	936.8	100.86	23.221	160.499
**VPG_6**	788.302	132.781	196.169	35.728	37.011	41.185	208.3	2528.822	939.028	98.216	22.756	157.202
**VPG_7**	670.062	107.558	148.449	23.137	36.75	52.249	156.032	2529.77	748.954	136.89	33.569	163.159
**VPG_8**	683.855	110.766	151.819	27.42	38.155	54.868	152.862	2370.571	757.561	139.77	33.724	155.594
**VPG_9**	639.876	111.716	152.934	25.426	36.654	50.791	156.025	2468.769	742.17	133.83	33.68	162.548
