# Supplementary material for: Insight into the Molecular Signature of Skeletal Muscle Characterizing Lifelong Football Players
Source: Int J Environ Res Public Health. 2022 Nov 28;19(23):15835. doi: 10.3390/ijerph192315835 (PMC9740844; doi:10.3390/ijerph192315835)
Supplement: Supplementary file 1 [file ijerph-19-15835-s001.zip › Table S3.pdf]

Table S3. Metabolite concentrations (microM) in muscle sample

	C0	C2	C3	C4	C5	C6	C8	C10	C12	C14	C16	C18
**CG_1**	1431.952	298.607	0.307	0.103	0.083	0.037	0.038	0.096	0.047	0.031	0.091	No data
**CG_2**	1803.842	403.057	0.398	0.051	0.047	0.028	0.034	0.103	0.083	0.033	0.052	0.035
**CG_3**	1633.107	301.742	0.389	0.073	0.085	0.074	0.049	0.065	0.037	0.037	0.058	0.029
**CG_4**	1794.908	256.983	0.078	0.048	0.023	No data	0.019	0.038	0.056	0.056	0.482	0.042
**CG_5**	1669.779	257.345	0.171	0.118	0.064	0.026	0.104	0.104	0.047	No data	0.058	0.102
**CG_6**	1734.021	227.098	0.648	0.059	0.03	0.05	0.054	0.054	0.094	0.079	0.035	0.017
**CG_7**	1600.631	91.826	0.168	0.05	0.04	0.008	0.01	0.085	0.023	0.023	0.054	0.027
**CG_8**	1705.103	86.322	0.26	0.049	0.037	0.037	0.03	0.05	0.038	0.028	0.071	0.036
**CG_9**	1805.944	93.945	0.476	0.042	0.022	0.077	0.05	0.025	0.03	0.04	0.019	0.019
**VPG_1**	1756.19	205.484	0.306	0.115	0.034	0.011	0.071	0.035	0.036	0.054	0.027	0.009
**VPG_2**	1756.958	202.576	0.382	0.045	0.04	0.02	0.046	0.046	0.017	0.026	0.01	0.03
**VPG_3**	2002.197	193.612	0.843	0.024	0.032	No data	0.037	0.05	0.032	0.011	0.051	0.031
**VPG_4**	1534.527	203.14	0.54	0.024	0.057	0.045	0.043	0.043	0.035	0.026	0.036	0.009
**VPG_5**	1654.274	208.31	0.278	0.115	0.022	0.011	0.085	0.146	0.049	0.039	0.017	0.035
**VPG_6**	1612.113	173.612	0.521	0.077	0.076	0.038	0.039	0.039	0.039	0.02	0.042	0.008
**VPG_7**	1733.324	234.918	0.667	0.036	0.049	0.016	0.077	0.058	0.025	0.038	0.011	0.022
**VPG_8**	1863.375	188.107	0.227	0.03	0.029	0.029	0.021	0.063	0.055	0.069	0.048	0.024
**VPG_9**	1732.522	200.478	0.298	0.11	0.052	0.026	0.03	0.06	0.071	0.024	0.044	0.022
