# Supplementary material for: Insight into the Molecular Signature of Skeletal Muscle Characterizing Lifelong Football Players
Source: Int J Environ Res Public Health. 2022 Nov 28;19(23):15835. doi: 10.3390/ijerph192315835 (PMC9740844; doi:10.3390/ijerph192315835)
Supplement: Supplementary file 1 [file ijerph-19-15835-s001.zip › Table S4.pdf]

Table S4 Unsaturated AC concentrations (microM) in muscle sample

	C5:1	C6:1	C8:1	C10:1	C10:2	C12:1	C14:1	C14:2	C16:1	C18:1	C18:2
**CG_1**	0.037	0.046	0.057	0.038	0.115	0.031	0.016	0.047	0.136	0.03	No data
**CG_2**	0.093	0.13	0.051	0.051	0.154	0.067	0.083	0.083	0.035	0.035	0.087
**CG_3**	0.042	0.064	0.114	0.033	0.065	0.037	0.018	0.037	0.029	0.029	0.087
**CG_4**	0.035	0.035	0.038	0.038	0.075	0.075	0.075	0.056	0.147	0.042	No data
**CG_5**	0.064	0.077	0.083	0.083	0.083	0.047	0.031	0.016	0.044	0.015	0.029
**CG_6**	0.05	0.05	0.054	0.054	0.036	0.047	0.016	0.094	0.104	0.052	0.087
**CG_7**	0.063	0.048	0.048	0.038	0.066	0.015	0.031	0.015	0.027	0.009	0.045
**CG_8**	0.019	0.065	0.05	0.02	0.099	0.066	0.038	0.009	0.027	0.045	0.018
**CG_9**	0.055	0.033	0.025	0.025	0.075	0.02	0.03	0.02	0.029	0.019	0.019
**VPG_1**	0.034	0.068	0.024	0.047	0.106	0.063	0.036	0.009	0.054	0.018	0.036
**VPG_2**	0.03	0.04	0.011	0.034	0.057	0.043	0.017	0.009	0.01	0.05	0.03
**VPG_3**	0.032	0.042	0.05	0.025	0.037	0.043	0.022	0.011	0.031	0.041	0.031
**VPG_4**	0.034	0.09	0.011	0.065	0.054	0.069	0.061	0.009	0.009	0.018	0.063
**VPG_5**	No data	0.043	0.049	0.049	0.036	0.039	0.069	0.02	0.026	0.017	0.043
**VPG_6**	0.013	0.05	0.052	0.078	0.065	0.039	0.069	0.02	0.008	0.025	0.017
**VPG_7**	0.066	0.016	0.019	0.058	0.058	0.063	0.025	0.013	0.011	0.022	0.022
**VPG_8**	0.058	0.101	0.084	0.063	0.084	0.014	0.028	0.028	0.024	0.036	0.012
**VPG_9**	0.039	0.105	0.06	0.045	0.075	0.012	0.047	0.035	0.033	0.011	0.011
