# Supplementary material for: Insight into the Molecular Signature of Skeletal Muscle Characterizing Lifelong Football Players
Source: Int J Environ Res Public Health. 2022 Nov 28;19(23):15835. doi: 10.3390/ijerph192315835 (PMC9740844; doi:10.3390/ijerph192315835)
Supplement: Supplementary file 1 [file ijerph-19-15835-s001.zip › Table S5.pdf]

Table S5. Branched AC concentrations (microM) in muscle sample

	C3DC	C4DC	C5DC	C6DC	C8DC	C10DC
**CG_1**	0.115	0.153	0.115	0.078	0.047	0.045
**CG_2**	0.068	0.154	0.12	0.05	0.033	0.069
**CG_3**	0.065	0.13	0.065	0.037	0.11	0.087
**CG_4**	0.038	0.075	0.3	0.038	0.094	0.252
**CG_5**	0.062	0.104	0.393	0.031	0.078	0.029
**CG_6**	0.054	0.108	0.288	0.047	0.063	0.052
**CG_7**	0.038	0.057	0.048	0.015	0.046	0.027
**CG_8**	0.069	0.079	0.069	0.009	0.019	0.027
**CG_9**	0.025	0.137	0.137	0.03	0.05	0.029
**VPG_1**	0.035	0.177	0.047	0.027	0.054	0.027
**VPG_2**	0.08	0.114	0.023	0.017	0.009	0.03
**VPG_3**	0.125	0.1	0.075	0.022	0.011	0.051
**VPG_4**	0.011	0.086	0.032	0.026	0.035	0.036
**VPG_5**	0.073	0.146	0.049	0.01	0.03	0.043
**VPG_6**	0.065	0.155	0.09	0.039	0.02	0.008
**VPG_7**	0.039	0.096	0.116	No data	0.025	0.055
**VPG_8**	0.106	0.084	0.021	0.041	0.055	0.024
**VPG_9**	0.045	0.045	0.03	0.059	0.012	0.056
