# Supplementary material for: Insight into the Molecular Signature of Skeletal Muscle Characterizing Lifelong Football Players
Source: Int J Environ Res Public Health. 2022 Nov 28;19(23):15835. doi: 10.3390/ijerph192315835 (PMC9740844; doi:10.3390/ijerph192315835)
Supplement: Supplementary file 1 [file ijerph-19-15835-s001.zip › Table S6.pdf]

Table S6. Hydroxylated AC concentrations (microM) in muscle sample

	C4OH	C5OH	C6OH	C12OH	C14OH	C16OH	C16:1OH	C18OH	C18:1OH
**CG_1**	0.083	0.037	0.083	0.016	0.076	0.045	0.076	No data	0.045
**CG_2**	0.019	0.084	0.065	0.017	0.139	0.087	0.087	0.035	0.017
**CG_3**	0.032	0.032	0.042	0.092	0.058	0.029	0.015	No data	0.029
**CG_4**	0.023	0.07	0.058	0.094	0.252	0.147	0.063	0.042	0.063
**CG_5**	0.026	0.038	0.128	0.062	0.276	0.102	0.015	0.044	0.015
**CG_6**	0.06	0.04	0.03	0.063	0.312	0.035	0.017	No data	0.017
**CG_7**	0.024	0.04	0.04	0.023	0.081	0.099	0.027	0.027	No data
**CG_8**	0.028	0.056	0.074	0.019	0.08	0.089	0.009	0.009	0.045
**CG_9**	0.022	0.077	0.033	0.02	0.058	0.106	0.039	No data	0.019
**VPG_1**	0.034	0.056	0.034	0.018	0.036	0.081	0.018	0.009	0.018
**VPG_2**	0.071	0.04	0.061	0.017	0.05	0.099	0.02	0.04	0.02
**VPG_3**	0.032	0.063	0.063	0.011	0.061	0.113	No data	0.021	0.01
**VPG_4**	0.079	0.023	0.113	0.035	0.054	0.045	0.018	0.018	0.018
**VPG_5**	0.054	0.043	0.054	0.03	0.095	0.035	0.009	0.009	No data
**VPG_6**	0.05	0.101	0.138	0.02	0.05	0.067	0.025	No data	0.017
**VPG_7**	0.033	0.082	0.066	0.025	0.033	0.044	No data	No data	0.011
**VPG_8**	0.043	0.043	0.029	0.014	0.024	0.084	0.036	No data	No data
**VPG_9**	0.039	0.052	0.039	0.012	0.044	0.056	0.011	0.011	0.044
